# Crystal structure of 1-(piperidin-1-yl)butane-1,3-dione

**DOI:** 10.1107/S1600536814025768

**Published:** 2014-11-29

**Authors:** Markus Schwierz, Helmar Görls, Wolfgang Imhof

**Affiliations:** a University Koblenz-Landau, Institute for Integrated Natural Sciences, Universitätsstrasse 1, 56070 Koblenz, Germany; b Friedrich-Schiller-University Jena, Institute of Inorganic and Analytical Chemistry, Humboldtstrasse 8, 07743 Jena, Germany

**Keywords:** crystal structure, 1-(piperidin-1-yl)butane-1,3-dione, weak hydrogen bonding

## Abstract

In the title compound, C_9_H_15_NO_2_, the piperidine ring exhibits a chair conformation. The butane­dione subunit exhibits a conformation with the ketone C atom in an eclipsed position with respect to the amide carbonyl group. In the crystal, a two-dimensional layered arrangement is formed by hydrogen bonds of the C—H⋯O type between the methyl group and the exocyclic methyl­ene unit as donor sites and the amide carbonyl O atom as the acceptor of a bifurcated hydrogen bond. These layers are oriented parallel to the *ab* plane.

## Related literature   

For the synthetic procedure, see: Sridharan *et al.* (2010 ▶). For a survey concerning weak hydrogen bonds, see: Desiraju & Steiner (1999 ▶).
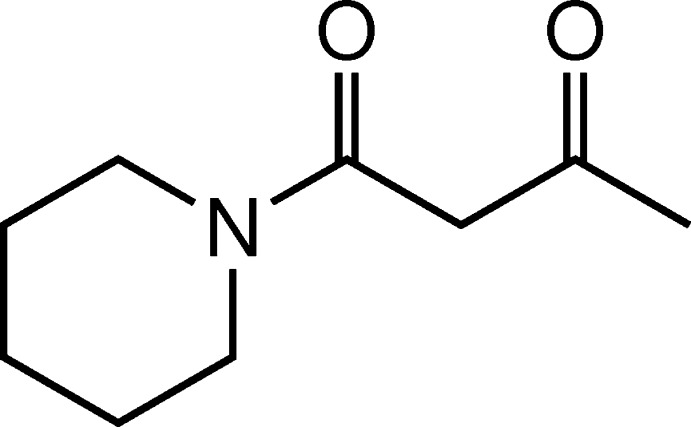


## Experimental   

### Crystal data  

C_9_H_15_NO_2_
*M* *_r_* = 169.22
Monoclinic, 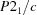

*a* = 5.4455 (1) Å
*b* = 9.1901 (2) Å
*c* = 17.8837 (4) Å
β = 94.506 (1)°
*V* = 892.22 (3) Å^3^
*Z* = 4
Mo *K*α radiation
μ = 0.09 mm^−1^
*T* = 133 K
0.12 × 0.09 × 0.07 mm

### Data collection  

Nonius KappaCCD diffractometer
Absorption correction: multi-scan (*SADABS*; Sheldrick, 2002 ▶) *T*_min_ = 0.717, *T*_max_ = 0.746
5612 measured reflections
2035 independent reflections
1777 reflections with *I* > 2σ(*I*)
*R*_int_ = 0.023

### Refinement  

*R*[*F* ^2^ > 2σ(*F* ^2^)] = 0.040
*wR*(*F* ^2^) = 0.107
*S* = 1.14
2035 reflections
169 parameters
All H-atom parameters refined
Δρ_max_ = 0.31 e Å^−3^
Δρ_min_ = −0.20 e Å^−3^

### 

Data collection: *COLLECT* (Nonius, 1998 ▶); cell refinement: *DENZO* (Otwinowski & Minor, 1997 ▶); data reduction: *DENZO* ; program(s) used to solve structure: *SHELXS97* (Sheldrick, 2008 ▶); program(s) used to refine structure: *SHELXL97* (Sheldrick, 2008 ▶); molecular graphics: *ORTEP-3 for Windows* (Farrugia, 2012 ▶) and *Mercury* (Macrae *et al.*, 2006 ▶); software used to prepare material for publication: *SHELXL97*.

## Supplementary Material

Crystal structure: contains datablock(s) I, New_Global_Publ_Block. DOI: 10.1107/S1600536814025768/bg2540sup1.cif

Structure factors: contains datablock(s) I. DOI: 10.1107/S1600536814025768/bg2540Isup2.hkl

Supporting information file. DOI: 10.1107/S1600536814025768/bg2540Isup3.cml

**Fig. 1.**
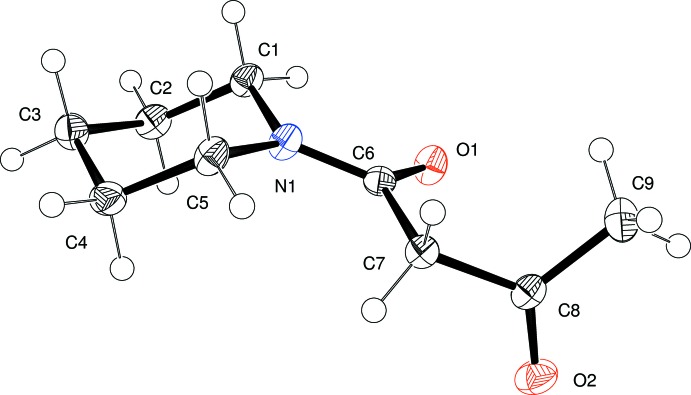
Molecular structure of the title compound with thermal ellipsoids drawn at the 50% probability level.

**Fig. 2.**
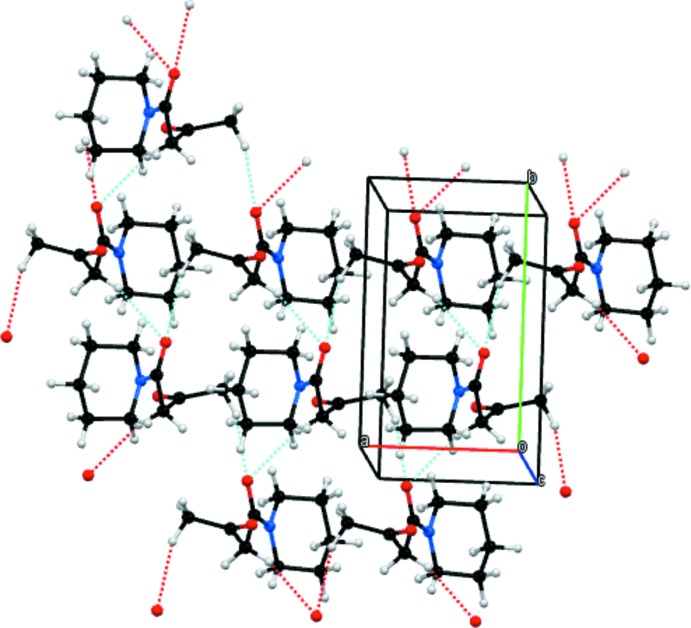
Crystal structure of the title compound showing layers of molecules along the ab plane which are built up by bifurcated C–H···O hydrogen bonds.

. DOI: 10.1107/S1600536814025768/bg2540fig1.tif
Mol­ecular structure of the title compound with thermal ellipsoids drawn at the 50% probability level.

ab . DOI: 10.1107/S1600536814025768/bg2540fig2.tif
Crystal structure of the title compound showing layers of mol­ecules along the *ab* plane which are built up by bifurcated C–H⋯O hydrogen bonds.

CCDC reference: 1035958

Additional supporting information:  crystallographic information; 3D view; checkCIF report

## Figures and Tables

**Table 1:** Hydrogen-bond geometry (, )

*D*H*A*	*D*H	H*A*	*D* *A*	*D*H*A*
C7H7*B*O1^i^	0.96(2)	2.459(17)	3.378(3)	161(1)
C9H9*B*O1^ii^	0.99(2)	2.601(18)	3.466(3)	146(1)

Symmetry codes: (i) 
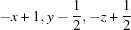
; (ii) 
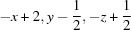
.

## supplementary crystallographic information

### S1. Comment

The title compound is an intermediate in the synthesis of 2,2-dimethoxy-1-(pyridin-2-yl)ethanone and has been synthesized from piperidine and 2,2,6-trimethyl-4*H*-1,3-dioxin-4-one following a modified procedure (Sridharan *et al.*, 2010). As it is expected the piperidine ring shows a chair conformation and the amide substructure is planar. The butanedione subunit exhibits a conformation with the ketone carbon atom in an eclipsed position with respect to the amide carbonyl group (Figure 1). The dihedral angle between the two carbonyl groups therefore measures to 81.4°. In the crystal structure, a two-dimensional layered arrangement is formed by hydrogen bonds of the C–H···O type between the methyl group and the exocyclic methylene unit as donor sites and the amide carbonyl oxygen atom as the acceptor of a bifurcated hydrogen bond. These layers are oriented along to the *ab* plane (Figure 2).

### S2. Experimental

0.2 Mol of piperidine (17.0 g, 19.8 ml), 0.2 mol of sodium acetate (17.0 g) and a slight excess (0.26 mol, 37.0 g, 34.6 ml) 2,2,6-trimethyl-4*H*-1,3-dioxin-4-one together with 40 ml THF are refluxed for 24 h. After cooling down to room temperature the solution is filtered and the remaining sodium acetate is washed with diethylether (3 × 20 ml). The combined THF and diethylether solutions are treated with brine (3 × 25 ml) and dried with Na_2_SO_4_. After filtration the organic solution is evaporated to dryness. The raw product may either be purified by chromatography (silica, light petroleum: ethyl acetate = 9: 1, yield 67%) or by destillation *in vacuo* (0.2 mbar, yield: 83%). If only the product fraction that is condensed into a Schlenk tube which is cooled with liquid nitrogen using a bath temperature above 100°C is collected, crystalline material of the title compound suitable for X-ray crystallography is obtained.

### S3. Refinement

The positions of all hydrogen atoms have been determined from a Fourier map and all hydrogen atoms were refined without any constraints.

### Crystal data

**Table:**

C_9_H_15_NO_2_	*Z* = 4
*M**_r_* = 169.22	*F*(000) = 368
Monoclinic, *P*2_1_/*c*	*D*_x_ = 1.260 Mg m^−^^3^
Hall symbol: -P 2ybc	Mo *K*α radiation, λ = 0.71073 Å
*a* = 5.4455 (1) Å	µ = 0.09 mm^−^^1^
*b* = 9.1901 (2) Å	*T* = 133 K
*c* = 17.8837 (4) Å	Prism, colourless
β = 94.506 (1)°	0.12 × 0.09 × 0.07 mm
*V* = 892.22 (3) Å^3^	

### Data collection

**Table:**

Nonius KappaCCD diffractometer	2035 independent reflections
Radiation source: fine-focus sealed tube	1777 reflections with *I* > 2σ(*I*)
Graphite monochromator	*R*_int_ = 0.023
phi– + ω–scan	θ_max_ = 27.5°, θ_min_ = 2.3°
Absorption correction: multi-scan (*SADABS*; Sheldrick, 2002)	*h* = −7→6
*T*_min_ = 0.717, *T*_max_ = 0.746	*k* = −7→11
5612 measured reflections	*l* = −23→21

### Refinement

**Table:**

Refinement on *F*^2^	Primary atom site location: structure-invariant direct methods
Least-squares matrix: full	Secondary atom site location: difference Fourier map
*R*[*F*^2^ > 2σ(*F*^2^)] = 0.040	Hydrogen site location: difference Fourier map
*wR*(*F*^2^) = 0.107	All H-atom parameters refined
*S* = 1.14	*w* = 1/[σ^2^(*F*_o_^2^) + (0.041*P*)^2^ + 0.4171*P*] where *P* = (*F*_o_^2^ + 2*F*_c_^2^)/3
2035 reflections	(Δ/σ)_max_ < 0.001
169 parameters	Δρ_max_ = 0.31 e Å^−^^3^
0 restraints	Δρ_min_ = −0.20 e Å^−^^3^

### Special details

**Table:**

Geometry. All e.s.d.'s (except the e.s.d. in the dihedral angle between two l.s. planes) are estimated using the full covariance matrix. The cell e.s.d.'s are taken into account individually in the estimation of e.s.d.'s in distances, angles and torsion angles; correlations between e.s.d.'s in cell parameters are only used when they are defined by crystal symmetry. An approximate (isotropic) treatment of cell e.s.d.'s is used for estimating e.s.d.'s involving l.s. planes.
Refinement. Refinement of *F*^2^ against ALL reflections. The weighted *R*-factor *wR* and goodness of fit *S* are based on *F*^2^, conventional *R*-factors *R* are based on *F*, with *F* set to zero for negative *F*^2^. The threshold expression of *F*^2^ > σ(*F*^2^) is used only for calculating *R*-factors(gt) *etc*. and is not relevant to the choice of reflections for refinement. *R*-factors based on *F*^2^ are statistically about twice as large as those based on *F*, and *R*- factors based on ALL data will be even larger.

### Fractional atomic coordinates and isotropic or equivalent isotropic displacement parameters (Å^2^)

**Table:**

	*x*	*y*	*z*	*U*_iso_*/*U*_eq_	
O1	0.73273 (18)	0.88779 (10)	0.27173 (5)	0.0232 (2)	
O2	0.6753 (2)	0.70560 (11)	0.11035 (5)	0.0279 (3)	
N1	0.5948 (2)	0.73198 (12)	0.35732 (6)	0.0202 (3)	
C1	0.5498 (3)	0.84895 (15)	0.41067 (7)	0.0210 (3)	
H1B	0.661 (3)	0.8308 (18)	0.4570 (9)	0.022 (4)*	
H1A	0.594 (3)	0.9416 (19)	0.3881 (9)	0.023 (4)*	
C2	0.2840 (3)	0.84538 (15)	0.43124 (8)	0.0236 (3)	
H2B	0.257 (3)	0.9213 (19)	0.4687 (10)	0.027 (4)*	
H2A	0.175 (3)	0.868 (2)	0.3865 (11)	0.032 (5)*	
C3	0.2232 (3)	0.69623 (16)	0.46223 (8)	0.0236 (3)	
H3A	0.331 (3)	0.6804 (19)	0.5103 (10)	0.027 (4)*	
H3B	0.049 (4)	0.6953 (19)	0.4749 (10)	0.032 (5)*	
C4	0.2753 (3)	0.57716 (16)	0.40620 (8)	0.0230 (3)	
H4B	0.166 (3)	0.588 (2)	0.3604 (10)	0.033 (5)*	
H4A	0.245 (3)	0.4812 (19)	0.4270 (9)	0.027 (4)*	
C5	0.5391 (3)	0.58557 (14)	0.38390 (8)	0.0212 (3)	
H5B	0.657 (3)	0.5641 (18)	0.4283 (9)	0.021 (4)*	
H5A	0.565 (3)	0.5139 (19)	0.3448 (9)	0.023 (4)*	
C6	0.6852 (2)	0.76255 (14)	0.29111 (7)	0.0170 (3)	
C7	0.7267 (2)	0.63574 (14)	0.23861 (7)	0.0180 (3)	
H7B	0.575 (3)	0.5836 (18)	0.2286 (9)	0.022 (4)*	
H7A	0.854 (3)	0.5704 (19)	0.2607 (9)	0.024 (4)*	
C8	0.8154 (2)	0.69439 (14)	0.16614 (7)	0.0190 (3)	
C9	1.0830 (3)	0.73141 (18)	0.16694 (9)	0.0270 (3)	
H9C	1.137 (4)	0.780 (2)	0.2127 (12)	0.046 (6)*	
H9B	1.177 (4)	0.639 (2)	0.1650 (12)	0.049 (6)*	
H9A	1.116 (4)	0.784 (2)	0.1229 (13)	0.053 (6)*	

### Atomic displacement parameters (Å^2^)

**Table:**

	*U*^11^	*U*^22^	*U*^33^	*U*^12^	*U*^13^	*U*^23^
O1	0.0316 (5)	0.0149 (5)	0.0240 (5)	0.0001 (4)	0.0072 (4)	0.0018 (4)
O2	0.0346 (6)	0.0292 (6)	0.0191 (5)	−0.0014 (4)	−0.0030 (4)	0.0028 (4)
N1	0.0295 (6)	0.0133 (5)	0.0183 (5)	−0.0003 (4)	0.0048 (4)	−0.0007 (4)
C1	0.0284 (7)	0.0164 (6)	0.0183 (6)	−0.0016 (5)	0.0035 (5)	−0.0030 (5)
C2	0.0285 (7)	0.0198 (7)	0.0226 (7)	0.0029 (5)	0.0024 (5)	−0.0021 (5)
C3	0.0231 (7)	0.0237 (7)	0.0244 (7)	−0.0008 (5)	0.0040 (5)	−0.0001 (5)
C4	0.0269 (7)	0.0208 (7)	0.0209 (7)	−0.0043 (5)	−0.0007 (5)	0.0003 (5)
C5	0.0309 (7)	0.0144 (6)	0.0188 (6)	−0.0006 (5)	0.0042 (5)	0.0020 (5)
C6	0.0167 (6)	0.0164 (6)	0.0176 (6)	0.0011 (5)	−0.0006 (5)	0.0005 (5)
C7	0.0200 (6)	0.0156 (6)	0.0183 (6)	0.0004 (5)	0.0015 (5)	0.0000 (5)
C8	0.0249 (7)	0.0142 (6)	0.0183 (6)	0.0023 (5)	0.0032 (5)	−0.0013 (5)
C9	0.0238 (7)	0.0298 (8)	0.0281 (8)	0.0013 (6)	0.0076 (6)	0.0033 (6)

### Geometric parameters (Å, º)

**Table:**

O1—C6	1.2353 (16)	C4—C5	1.523 (2)
O2—C8	1.2117 (17)	C4—H4B	0.980 (18)
N1—C6	1.3472 (17)	C4—H4A	0.976 (18)
N1—C5	1.4667 (16)	C5—H5B	1.001 (16)
N1—C1	1.4706 (16)	C5—H5A	0.978 (17)
C1—C2	1.521 (2)	C6—C7	1.5244 (18)
C1—H1B	1.001 (16)	C7—C8	1.5171 (18)
C1—H1A	0.981 (17)	C7—H7B	0.959 (17)
C2—C3	1.525 (2)	C7—H7A	0.976 (17)
C2—H2B	0.987 (18)	C8—C9	1.496 (2)
C2—H2A	0.981 (19)	C9—H9C	0.96 (2)
C3—C4	1.525 (2)	C9—H9B	0.99 (2)
C3—H3A	1.012 (17)	C9—H9A	0.95 (2)
C3—H3B	0.993 (19)		
			
C6—N1—C5	125.13 (11)	H4B—C4—H4A	107.2 (14)
C6—N1—C1	120.59 (11)	N1—C5—C4	110.79 (11)
C5—N1—C1	114.27 (10)	N1—C5—H5B	107.6 (9)
N1—C1—C2	110.57 (11)	C4—C5—H5B	110.1 (9)
N1—C1—H1B	107.1 (9)	N1—C5—H5A	110.0 (10)
C2—C1—H1B	108.7 (9)	C4—C5—H5A	110.2 (9)
N1—C1—H1A	108.0 (10)	H5B—C5—H5A	108.2 (13)
C2—C1—H1A	112.9 (9)	O1—C6—N1	122.69 (12)
H1B—C1—H1A	109.4 (13)	O1—C6—C7	119.67 (12)
C1—C2—C3	110.19 (11)	N1—C6—C7	117.64 (11)
C1—C2—H2B	110.2 (10)	C8—C7—C6	109.09 (10)
C3—C2—H2B	109.9 (10)	C8—C7—H7B	110.2 (10)
C1—C2—H2A	108.8 (11)	C6—C7—H7B	109.2 (10)
C3—C2—H2A	110.9 (11)	C8—C7—H7A	107.6 (10)
H2B—C2—H2A	106.8 (14)	C6—C7—H7A	110.8 (10)
C2—C3—C4	110.44 (12)	H7B—C7—H7A	109.9 (14)
C2—C3—H3A	108.1 (10)	O2—C8—C9	122.56 (13)
C4—C3—H3A	109.2 (10)	O2—C8—C7	120.78 (12)
C2—C3—H3B	109.2 (10)	C9—C8—C7	116.62 (12)
C4—C3—H3B	111.9 (10)	C8—C9—H9C	110.5 (13)
H3A—C3—H3B	107.8 (14)	C8—C9—H9B	108.1 (12)
C5—C4—C3	111.32 (11)	H9C—C9—H9B	107.5 (18)
C5—C4—H4B	107.5 (11)	C8—C9—H9A	110.6 (13)
C3—C4—H4B	110.3 (11)	H9C—C9—H9A	113.9 (18)
C5—C4—H4A	109.8 (10)	H9B—C9—H9A	105.9 (18)
C3—C4—H4A	110.6 (10)		

### Hydrogen-bond geometry (Å, º)

**Table:**

*D*—H···*A*	*D*—H	H···*A*	*D*···*A*	*D*—H···*A*
C7—H7*B*···O1^i^	0.96 (2)	2.459 (17)	3.378 (3)	161 (1)
C9—H9*B*···O1^ii^	0.99 (2)	2.601 (18)	3.466 (3)	146 (1)

Symmetry codes: (i) −*x*+1, *y*−1/2, −*z*+1/2; (ii) −*x*+2, *y*−1/2, −*z*+1/2.
