# Supplementary figures and images for: Crystal structure of 1-(piperidin-1-yl)butane-1,3-dione
Source: Acta Crystallogr Sect E Struct Rep Online. 2014 Nov 29;70(Pt 12):o1297. doi: 10.1107/S1600536814025768 (PMC4257425; doi:10.1107/S1600536814025768)

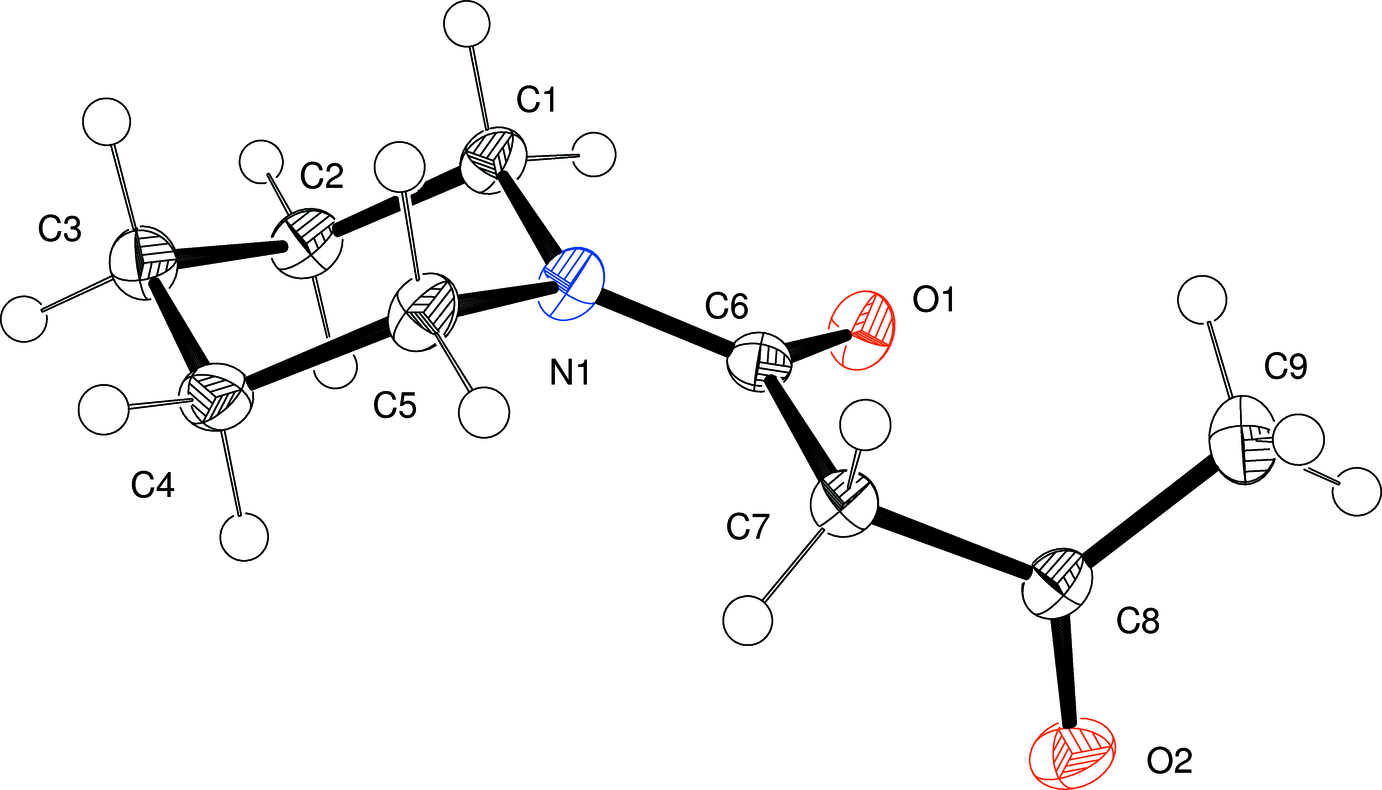

Supplement: Supplementary file 4 [file e-70-o1297-fig1.tif]

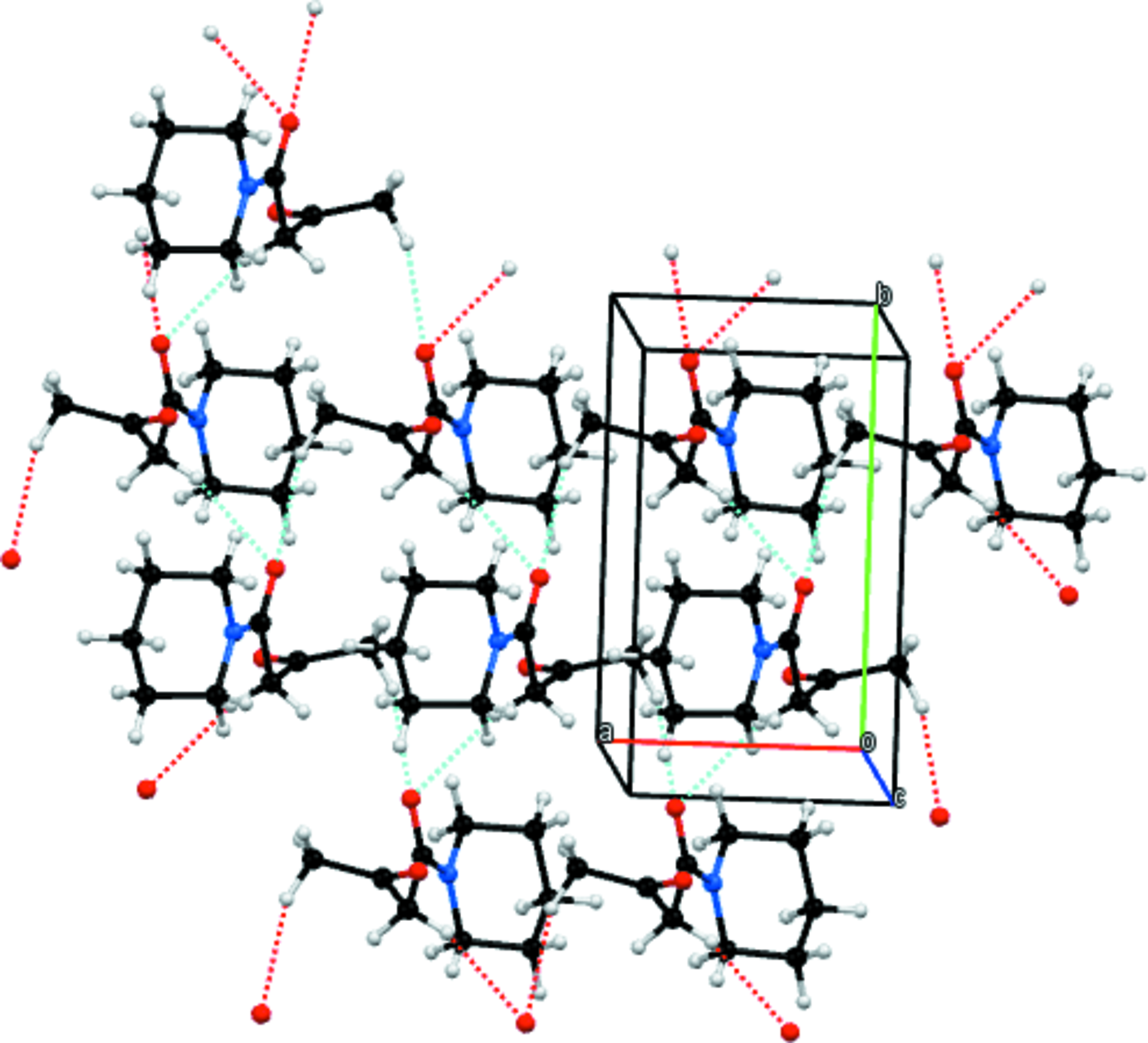

Supplement: Supplementary file 5 [file e-70-o1297-fig2.tif]
